# Corneal nerves and their role in dry eye pathophysiology

**DOI:** 10.1016/j.exer.2022.109191

**Published:** 2022-07-16

**Authors:** Alexia Vereertbrugghen, Jeremías G. Galletti

**Affiliations:** Innate Immunity Laboratory, Institute of Experimental Medicine (IMEX), CONICET- National Academy of Medicine, Buenos Aires, Argentina

**Keywords:** Ocular surface, Corneal sensitivity, Neurobiology, Pain, dry eye, Corneal nerves

## Abstract

As the cornea is densely innervated, its nerves are integral not only to its structure but also to its pathophysiology. Corneal integrity depends on a protective tear film that is maintained by corneal sensation and the reflex arcs that control tearing and blinking. Furthermore, corneal nerves promote epithelial growth and local immunoregulation. Thus, corneal nerves constitute pillars of ocular surface homeostasis. Conversely, the abnormal tear film in dry eye favors corneal epithelial and nerve damage. The ensuing corneal nerve dysfunction contributes to dry eye progression, ocular pain and discomfort, and other neuropathic symptoms. Recent evidence from clinical studies and animal models highlight the significant but often overlooked neural dimension of dry eye pathophysiology. Herein, we review the anatomy and physiology of corneal nerves before exploring their role in the mechanisms of dry eye disease.

## 1 Introduction

The cornea has the highest density of innervation in the human body (Rózsa and Beuerman, 1982), and thus, corneal nerves are inherently intertwined not only with corneal structure and physiology but also with corneal pathophysiology. First, corneal health requires the protection from desiccation afforded by the tear film(Willcox et al., 2017). The maintenance of an adequate tear film depends on corneal nerves because the reflex arcs that control tearing and blinking rely on sensory input from the ocular surface. Second, corneal nerves contribute to corneal epithelial integrity and local immune regulation(Galletti and de Paiva, 2021a; Sabatino et al., 2017). Thus, corneal nerves constitute pillars of ocular surface homeostasis. Conversely, dry eye disease (DED) is a highly prevalent disorder in which the cornea is affected due to tear film abnormalities(Willcox et al., 2017). The ensuing corneal nerve damage and dysfunction favor disease progression and contribute to one of the key symptoms of dry eye, ocular pain. Therefore, corneal nerves are involved in several aspects of DED pathophysiology and mounting evidence adds a whole new neural dimension to our understanding of the mechanisms of the disease. There are already excellent reviews on corneal nerves but their level of detail may be daunting to non--neuroscientists(Al-Aqaba et al., 2019, 2010; Marfurt et al., 2010). Consequently, we reviewed the anatomy and physiology of corneal innervation in humans and animals before exploring its role in DED pathophysiology, and we did so with a non-neuroscientist reader in mind, introducing neural concepts as they are mentioned.

## 2 Anatomy of corneal innervation

The cornea receives both sensory and autonomic innervation. Sensory nerves relay information to the central nervous system after sensory transduction through different types of receptors, while autonomic nerves control multiple aspects of corneal physiology(McDougal and Gamlin, 2015; Xue et al., 2018). Corneal sensory innervation is more abundant than its autonomic counterpart, and it is supplied by the ophthalmic branch (V1) of the trigeminal nerve (V cranial nerve), which carries the projections from 50 to 450 pseudounipolar neurons whose cell bodies are located in the trigeminal ganglion (2% of total trigeminal neurons) (Krastev and Apostolov, 2013; Belmonte et al., 2017). Pseudounipolar refers to the anatomy of the neuron: only one projection leaves the cell body but then divides into two axons, one of which travels to the brainstem while the other proceeds to the eye(Krastev and Apostolov, 2013). Autonomic innervation reaches the eye differently depending on whether it is sympathetic or parasympathetic(McDougal and Gamlin, 2015). Corneal autonomic innervation is predominantly sympathetic and represents at most 10–15% of corneal nerve fibers (Marfurt et al., 1989). In fact, parasympathetic corneal innervation in the human cornea has not been confirmed(Al-Aqaba et al., 2019; Müller et al., 2003). Thus, corneal innervation comprises the sensory axons of trigeminal neurons and the sympathetic autonomic axons from post-ganglionic neurons in the superior cervical ganglion, all of which traverse the suprachoroidal space through the short and long ciliary nerves to reach the limbus, where they give rise to the limbal plexus(Al-Aqaba et al., 2019). From there, nerves emerge radially and enter the cornea, where they shed their perineurium (protective sheath of connective tissue that surrounds a nerve) and myelin sheath shortly after crossing the limbus and continue as stromal nerves evenly distributed along the corneal circumference (~70 and ~40 stromal bundles in the human and murine cornea, respectively) (Al-Aqaba et al., 2019; Marfurt et al., 2010; Müller et al., 2003). Therefore, in the center and most of the periphery, stromal nerves are surrounded only by Schwann cells and are unmyelinated, which is required for corneal transparency(Al-Aqaba et al., 2019; Müller et al., 2003). In the peripheral cornea, stromal nerves are found in the middle third of the stroma, but they move anteriorly as they travel towards the center. Contrastingly, the posterior stroma is scarcely innervated.

**Fig. 1:**
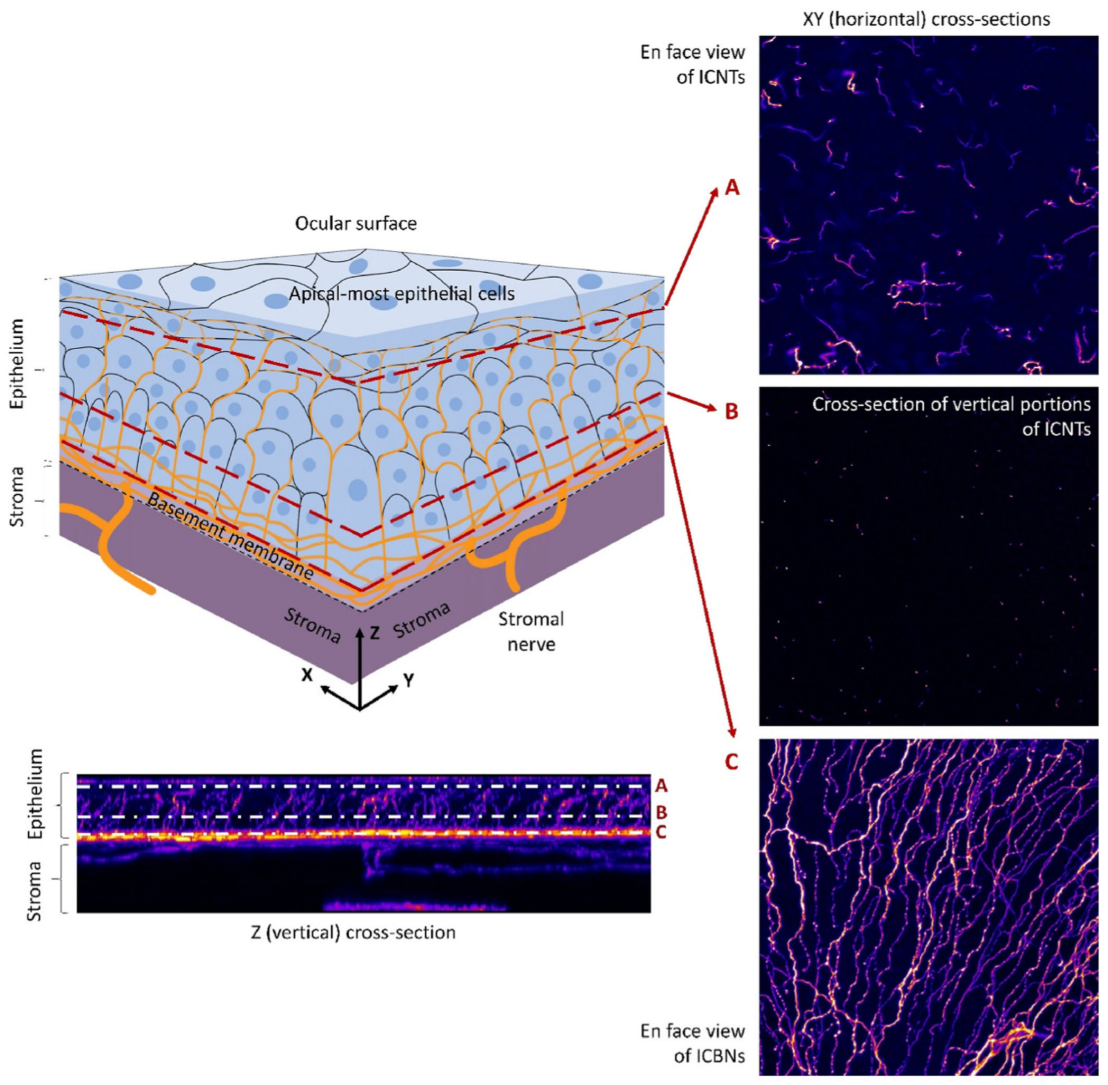
Intraepithelial corneal nerve anatomy. Schematic diagram of intraepithelial corneal nerves (top left) and representative confocal microscopy images of a vertical (Z, bottom) and horizontal (XY, right) cross-sections taken at 3 different depths (A, B, C) within the epithelium of a corneal wholemount (Balb/c mouse) labeled with anti-tubulin β3 antibody (TUJ1 clone).

Starting at the periphery of the cornea, close to the limbus, the stromal bundles branch and penetrate Bowman’s layer and the epithelial basement membrane orthogonally and enter the corneal epithelium (Stepp et al., 2017). Then, these nerve fibers take a 90° turn and extend towards the central cornea, traveling between the epithelial basement membrane and the basal epithelium while continuing to branch out and connect, thereby forming the so-called subbasal plexus(He et al., 2010). The term subbasal is widely used in the literature but it does not accurately describe the exact location of these nerves (Fig. 1): they are sheltered within infoldings of the basal cell membrane of basal epithelial cells(Al-Aqaba et al., 2010). Instead, the term intraepithelial corneal basal nerves (ICBNs) is more appropriate and we will use this new nomenclature proposed by Stepp et al. throughout this review(Stepp et al., 2020a). ICBNs, in turn, give rise to intraepithelial corneal nerve terminals (ICNTs), which end near the apical cells of the corneal epithelium. ICBNs and ICNTs are the two truly intraepithelial components of the corneal nerves and are collectively referred to as intraepithelial corneal nerves (ICNs) (Stepp et al., 2020a).

ICN anatomy is unique in several ways (Fig. 1). First, ICBNs can be several millimeters long (human corneal diameter: ~11 mm) (Downie et al., 2021) but they lose glial support as they enter the epithelial layer. Instead of glial cells, the basal membrane infoldings of corneal basal epithelial cells provide support to ICBNs as do Schwann cells elsewhere (Stepp et al., 2017). Thus, there is extensive contact between ICBNs and epithelial cells, and even scattered focal fusion of cell membranes between corneal axons and basal epithelial cells has been reported in mice (Courson et al., 2019). Second, as ICBNs converge centripetally, they form a characteristic whorl pattern(Al-Aqaba et al., 2010; Patel and McGhee, 2005) that is centered inferior to the corneal apex(Patel and McGhee, 2008). This whorl pattern mirrors that of corneal epithelial cells migrating from the limbus towards the corneal center(Di Girolamo, 2015). Since corneal epithelial cells appear to follow electromagnetic cues from the eye dipole(Patel and McGhee, 2005), the matching pattern of the intraepithelial corneal nerves probably results from the intricate interactions between the two(Al-Aqaba et al., 2019; Di Girolamo, 2015). Third, ICBNs branch continuously along their course, giving rise to ICNTs that may run horizontally at first and then turn vertically towards the apical cell layer. As the ICNTs encounter the tight junctions surrounding the apical-most epithelial squames, they are forced to turn parallel to the ocular surface and they continue to grow. As a result, when viewed *en face* from above, the ICNTs appear more abundant apically than they do in the mid-epithelium where they are imaged in cross-section only (Fig. 1). Fourth, ICNTs are classified according to their branching pattern as simple, ramifying, or complex. Simple endings do not branch once they leave the intraepithelial corneal plexus, whereas ramifying endings divide only once, right beneath the superficial squamous epithelial cells, into a leash of 3–4 endings. By contrast, complex endings branch several times, starting at the wing cell layer(Belmonte et al., 2017). All types of ICNTs end with a bulbous swelling within or below the superficial squamous cells(Al-Aqaba et al., 2019). Thus, ICNTs are almost in direct contact with the tear film and adequately posed to sense the ocular surface microenvironment (Section 3). Conversely, in DED the ICNs are exposed to the dysfunctional tear film and as a consequence, they undergo morphological and functional changes (Section 4).

There are conflicting reports on ICBN density in humans. Some in vivo confocal microscopy studies of corneal nerves in patients showed that ICBN density decreases with age(Parissi et al., 2013; Roszkowska et al., 2021), while others did not observe this effect(Chirapapaisan et al., 2021; Gambato et al., 2015). In addition, none of these studies reported an effect of sex on ICBN density in humans. By contrast, mouse studies show a significant influence of age, sex, and strain on ICN morphology(Pham et al., 2019; Stepp et al., 2018a, 2020b). Probably, the reduced variability in inbred mice and other methodological differences facilitate the detection of the effects of age and gender on ICN morphology. Once confounding factors are considered, ICN morphological analysis by this method shows potential for early diagnosis of ocular and systemic disorders(Cruzat et al., 2017).

## 3 Physiology of corneal innervation

Corneal nerves serve numerous physiological functions that are essential to corneal homeostasis. Foremost is their somatosensory role, i. e., the ability to detect changes in the environment and the ocular surface itself and then relay the corresponding signals to diverse neural circuits so that appropriate responses take place. Among these responses, pain and its corresponding nociceptive behavior, regulation of tear production, and blinking are highly relevant in DED pathophysiology. In addition to these traditionally ascribed functions, corneal nerves also contribute to corneal epithelial homeostasis, the coordination of the immune response against threats, and local immunoregulation. For this latter aspect of corneal nerve physiology, we refer the reader to other reviews(Galletti and de Paiva, 2021a, 2021b; Müller et al., 2003; Sabatino et al., 2017).

### 3.1 Corneal nerve fibers and receptors

The cornea is innervated by C and Aδ fibers. C fibers (70–80% of corneal axons) are beaded (varicose), unmyelinated, and are usually bundled together by non-myelinating Schwann cells. C fibers have lower conduction velocity and sense painful, thermal, and chemical stimuli (Belmonte et al., 2017). They are also involved in neuropathic pain, i.e., pain caused by a lesion or disease of corneal nerves themselves(Ueda, 2008). By contrast, Aδ fibers (20–30% of corneal axons) are non-beaded (straight), thinly myelinated, wider, and have faster conduction velocity. These fibers are associated with acute pain and withdrawal reflexes (Belmonte et al., 2017). Notably, the dense corneal innervation does not interfere with light transmission, allowing the cornea to function correctly. This is because nerve bundles lose their myelin sheath shortly after entering the cornea at the limbus and become transparent(Al-A-qaba et al., 2010; He et al., 2010; Marfurt et al., 2010; Müller et al., 2003).

Based on their electrophysiological and molecular characteristics, corneal nerve fibers can be classified into three groups: polymodal nociceptors (respond to several noxious stimuli), cold thermoreceptors, and selective mechano-nociceptors(Belmonte, 2019; Belmonte et al., 2017). Many corneal nerve fibers are polymodal nociceptors: they are activated by mechanical energy, heat, chemical irritants, acidic pH, and endogenous ligands that are released upon tissue damage and inflammation(Belmonte et al., 2017). The transient receptor potential vanilloid 1 (TRPV1) ion channel is considered the molecular marker of polymodal nociceptor C fibers: it is directly activated by capsaicin, low pH, heat, and hyperosmolarity(Belmonte et al., 2017; Guzmán et al., 2020). As it is also activated indirectly by several endogenous inflammatory factors, it is regarded as the “pain transducer molecule.” (Belmonte, 2019; Julius and Basbaum, 2001) Capsaicin-mediated triggering of TRPV1 causes corneal pain in humans, and TRPV1 activation by inflammation increases the excitability of polymodal nociceptor fibers to other agonists (Immke and Gavva, 2006). Of note, corneal polymodal nociceptors can be sensitized by repeated stimulation, which may be of pathophysiological relevance(M. C. Acosta et al., 2013). Other channels found on polymodal nociceptors are the transient receptor potential ankyrin 1 (TRPA1) and the acid-sensing ion channel family(Callejo et al., 2015). TRPA1 is activated by a wide assortment of chemical irritants and endogenous inflammatory mediators(Bautista et al., 2013) and it is also involved in the sensitization of polymodal nociceptors but to a lesser extent than TRPV1(M. C. Acosta et al., 2013). In mice, TRPV1-expressing axons are C fibers that end within the superficial squamous corneal epithelial cells, whereas TRPA1 expression is restricted to Aδ fibers that end within the corneal stroma and basal epithelial cells(Alamri et al., 2015; Schecterson et al., 2020). However, functional studies of murine corneas suggest that some polymodal nociceptors coexpress TRPV1 and TRPA1(González-González et al., 2017). By contrast, corneal cold thermoreceptors are C fibers that end within the apical and wing corneal epithelial cells(Schecterson et al., 2020) and express transient receptor potential melastatin 8 (TRPM8), an ion channel activated by cooling, menthol, and osmolarity(Parra et al., 2010; Quallo et al., 2015). Thus, TRPM8 activation by changes in local temperature and/or tear osmolarity regulates eye blinking and ocular surface wetness(Parra et al., 2010; Quallo et al., 2015). Corneal cold thermoreceptors can be functionally divided into two different populations(González-González et al., 2017). On the one hand, low-threshold/high-activity cold thermoreceptors make up about ^2^/_3_ of corneal cold thermoreceptors, express higher TRPM8 levels, and exhibit continuous activity at the physiological corneal temperature (34–35 °C) that increases upon small temperature drops (≤0.5 °C) (Alcalde et al., 2018; Belmonte et al., 2017; González-González et al., 2017). On the other hand, high-threshold/low-activity cold thermoreceptors do not fire at normal corneal temperatures and require stronger cooling stimulation to become activated (<30 °C); they represent about ^1^/_3_ of corneal cold thermoreceptors, display lower TRPM8 levels, and probably coexpress TRPV1(Alcalde et al., 2018; Belmonte et al., 2017; González-González et al., 2017). Finally, there are selective mechano-nociceptors which are Aδ fibers that typically express Piezo2, a mechanically activated ion channel(Fernández-Trillo et al., 2020). They are responsible for sharp, acute corneal pain and the accompanying withdrawal reflex, and they might also be susceptible to sensitization under pathological circumstances(M. C. Acosta et al., 2013). Piezo2 expression is limited mostly to ICBNs(Fernández-Trillo et al., 2020).

There is a widespread notion in the literature that polymodal receptors amount to 70% of corneal nerves while cold thermoreceptors and selective mechano-nociceptors represent the remaining 10–15% and 20–30%, respectively(Belmonte et al., 2017; Downie et al., 2021). However, it should be noted that there are considerable discrepancies among individual studies, probably due to species-specific and methodological differences, and thus it would be premature to present unifying figures at this time. Guerrero-Moreno et al. reviewed these findings in detail(Guerrero-Moreno et al., 2020) and Table 1 summarizes key studies on the subject. For instance, a functional survey of corneal sensory terminals in the mouse cornea yielded a somewhat different picture: 10% of sensory terminals were pure mechanoreceptors; 40% were polymodal nociceptors that responded to a mechanical stimulus, heat, and capsaicin (a TRPV1 agonist), half of which (20% of total) also responded to a TRPA1 ligand; and the remaining 50% were cold ther-moreceptors(González-González et al., 2017). Consistent with this, in another mouse study 40% of corneal intraepithelial nerve fibers were TRPV1+, but this proportion increased to 80% for corneal stromal nerves(Jiao et al., 2021). Of note, a different study reported that half of the TRPM8+ corneal neurons in the trigeminal ganglion of mice coexpress TRPV1(Li et al., 2019). However, an independent survey of corneal axon terminals found no coexpression of TRPV1, TRPA1, and TRPM8 (Schecterson et al., 2020). Different findings were reported for the guinea pig cornea, where retrograde tracing revealed that 43% of corneal afferent neurons in the trigeminal ganglion express TRPV1, 8% express TRPM8, and 28% express Piezo2(Alamri et al., 2015). No coexpression of TRPV1 and Piezo2 was observed in these neurons, contrasting with some coexpression of TRPV1 and TRPM8. Remarkably, 20% of corneal afferent neurons in this study did not express any of the three channels, which suggests that they could represent silent nociceptors, that is, neurons that upregulate channel expression and become sensitive to stimuli under inflammatory conditions(Prato et al., 2017). It is also possible that there are still some unidentified corneal nerve fiber populations.

Finally, it should be mentioned that corneal nerve physiology is not static. Corneal mechanical sensitivity, an indicator of corneal nerve function, decreases with age(Mirzajan et al., 2015; Roszkowska et al., 2004) and is also possibly influenced by sex(Khezri et al., 2015; Roszkowska et al., 2004). This is in line with similar effects of age and sex on ICN morphology (see the previous section). Consistently, animal studies show that ion channel expression or the response patterns of corneal nerves change with age(Alcalde et al., 2018), inflammation(Masuoka et al., 2020), and in response to injury(Jiao et al., 2021), a fact that becomes highly relevant in DED pathophysiology.

### 3.2 Corneal nerves, tear production, and eye blinking

The tear film is formed by the concerted contribution of the meibomian glands, the lacrimal glands, goblet cells, and conjunctival and corneal epithelial cells, all of which exhibit specific secretory activity that is regulated by different neural mechanisms(Belmonte et al., 2017; Downie et al., 2021). The tear film that covers the ocular surface is thinnest (2 μm) over the cornea (the precorneal tear film) (Chen et al., 2010), where it is also the most exposed and susceptible to evaporation (Willcox et al., 2017). In the open eye, evaporation of water from the precorneal tear film leads to local tear hyperosmolarity until the tear film is replenished by a blink, initiating a new cycle(Willcox et al., 2017). Throughout this process, corneal nerves are involved in the two neural arcs that regulate tear production and blinking.

Most of the tear volume comes from the lacrimal glands, which continuously secrete at a basal rate but can also increase their output in response to sensory stimuli or emotions(Murube, 2009). This secretory activity is controlled by a reflex arc that combines sensory trigeminal innervation of the cornea in the afferent arm and autonomic innervation of the lacrimal gland through the facial nerve in the efferent arm(Meng and Kurose, 2013; Murube, 2009). Basal tear production relies mostly on continuous sensory input from the ocular surface, and accessorily, from the nasal mucosa (nasolacrimal reflex(Park et al., 2019), beyond the scope of this review). As the precorneal tear film thins and evaporates between blinks, the humidity of the corneal surface is sensed indirectly by its nerve endings through different mechanisms. Foremost, evaporation-induced cooling of the corneal surface leads to increased TRPM8 activation (Section 3.1), which also occurs in cold environments and increases basal tear production(Hirata and Oshinsky, 2012; Parra et al., 2010). However, drying of the corneal surface also activates cold-insensitive sensory neurons(Hirata and Oshinsky, 2012), which likely correspond to TRPV1-expressing polymodal receptors(Meng and Kurose, 2013). Both TRPV1 and TRPM8 ion channels respond to hyperosmolar conditions in the cornea and elsewhere(Lee et al., 2020; Pan et al., 2011; Quallo et al., 2015; Sharif Naeini et al., 2006), and there is conflicting evidence regarding the relative contribution of each receptor in vivo. TRPM8 is believed to be activated by small physiological fluctuations in tear osmolarity whereas TRPV1 activation might become more relevant at noxious osmolarity levels(Quallo et al., 2015). In the cornea, cold thermoreceptors fire spontaneously under physiological conditions, as opposed to polymodal nociceptors and mechanoreceptors (Gallar et al., 1993). TRPM8-deficient mice have reduced basal tearing but conserved irritation-induced tearing(Parra et al., 2010). Consistently, cold thermoreceptor activation increases tear production but is not associated with ocular pain, whereas mechanoreceptor and polymodal receptor activation is accompanied by nociceptive behavior in animals(Meng and Kurose, 2013; Robbins et al., 2012).

Spontaneous blinking, as opposed to reflex and voluntary blinking, is controlled by a still uncharacterized neural circuit known as the spontaneous blink generator(Kaminer et al., 2011). Its activity is modulated not only by input from corneal sensory innervation but also by cognitive processes, highlighting the complexity of the system(Doughty, 2001; Kaminer et al., 2011). Regarding the contribution of corneal sensory innervation to blinking, there is significant overlap with that of tear production, possibly because they are both protective mechanisms of the ocular surface. As such, cooling- and hyperosmolarity-induced TRPM8 activation of corneal cold thermoreceptors also regulates the spontaneous blinking rate(Quallo et al., 2015). However, the biphasic response of blinking rate to tear hyperosmolarity and/or ocular desiccation shows that TRPM8-independent, high-threshold corneal afferent input also plays a role(Hirata et al., 2018; Quallo et al., 2015). Thus, as is the case for tear secretion, the regulation of blinking similarly involves the detection of innocuous and nociceptive stimuli by corneal cold thermoreceptors and polymodal nociceptors, respectively.

In summary, sensory inflow from corneal nerves constitutes the afferent arm of two reflex arcs that are pillars of tear film homeostasis. It follows that by impairing these protective mechanisms, corneal nerve dysfunction might contribute to DED pathophysiology.

## 4 Corneal nerves and dry eye pathophysiology

### 4.1 Morphological changes in corneal nerves

The advent of in vivo confocal microscopy allowed for the non-invasive study of corneal nerves in patients. Since then, considerable evidence has accrued on the changes in corneal nerve morphology that accompany DED[see reviews (Alhatem et al., 2012; Cruzat et al., 2017; Guerrero-Moreno et al., 2020; Labetoulle et al., 2018)]. Most studies show a reduction in ICN density that correlates with disease severity, although there are some discrepancies between reports that probably reflect methodological differences(Cruzat et al., 2017). Both Sjögren’s and non-Sjögren’s aqueous deficient-DED patients have decreased ICN density(Labbé et al., 2013; Tepelus et al., 2017), which improves with topical cyclosporine A treatment(Levy et al., 2017). Other morphological changes in ICNs reported for DED are reduced thickness, hyper-reflectivity, and increased beading, tortuosity, and sprouting(Alhatem et al., 2012; Cruzat et al., 2017). The presence of corneal microneuromas as the result of stunted or abnormal nerve regeneration has also been associated with neuropathic corneal pain in DED(Dermer et al., 2021; Moein et al., 2020). However, the actual identity of these findings and whether they represent physiological or pathological structures is under question(Chinnery et al., 2021; Dermer et al., 2021; Stepp et al., 2020a). Of note, reduced corneal nerve density in DED patients is associated with a decreased response to anti-inflammatory treatment(Kheirkhah et al., 2015).

**Fig. 2:**
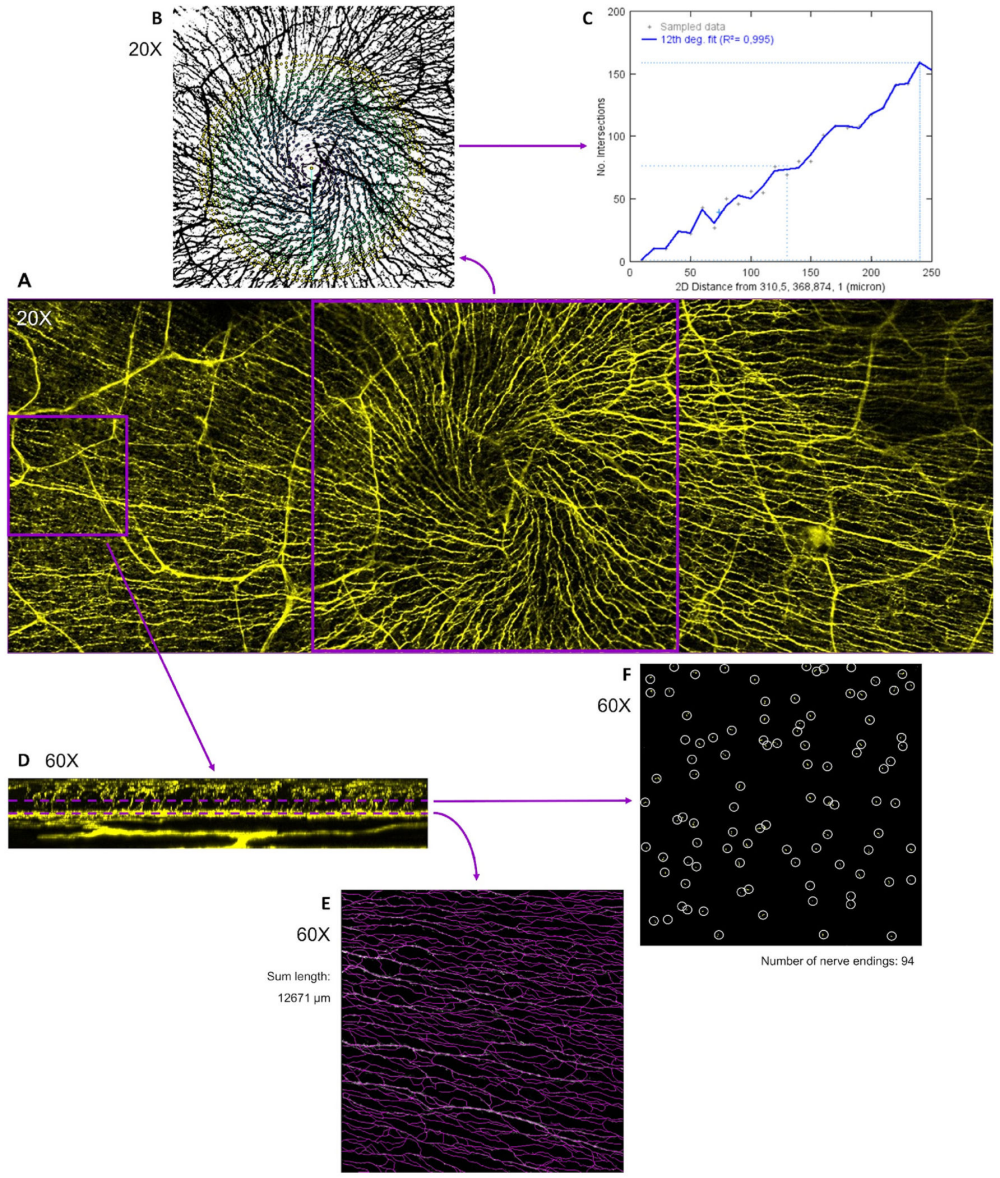
Quantitative analysis of corneal nerve morphology. Representative example of a murine corneal wholemount in which nerves were stained with anti-tubulin β3 antibody (TUJ1 clone) and the tissue was sequentially scanned in Z-stacks of 0.5–1 μm step size spanning the corneal epithelium and the anterior stroma (panel A). A 20X Z-projection centered on the nerve whorl (panel B) can be quantified by Sholl analysis (panel C), which counts the intersections of the branching nerves with concentric circles of increasing radii to create a profile (intersections over distance from center). Higher magnification (60X) scans obtained one 20X field away from the whorl (panel D) can be quantified at different depths. At the level of the intraepithelial corneal basal nerve plexus(panel E), nerves can be traced and the total nerve length measured (in μm/area), while at the intraepithelial nerve terminal level (panel F), the cross-sections of the terminals can be counted (number/examined area).

Mouse studies of different induced DED models report similar changes in ICN morphology (decreased ICBN and ICNT density). Fig. 2 shows the quantification methods used in these studies. These changes become evident early in the course of the disease (3–5 days, depending on the actual model) and develop simultaneously to corneal epithelial damage(Simsek et al., 2018; Stepp et al., 2018a). Acute corneal exposure to TRPM8 and TRPV1 ligands in rats also reduced ICN density without apparently affecting the corneal epithelium(Hegarty et al., 2017). We observed comparable alterations in ICN morphology in mice exposed to hyperosmolar stress for 5 days that did not have overt corneal epitheliopathy, highlighting the early onset of these changes(Guzmán et al., 2020). One study of a chronic induced DED model found increased tortuosity and hyperreflectivity of ICNs that were not observed in a different acute model, suggesting that these specific changes arise with nerve regeneration(Simsek et al., 2019). Surgical DED models created by lacrimal gland excision exhibited similar findings(Fakih et al., 2019; Yamazaki et al., 2017). Furthermore, decreased ICBN density that correlated with increased corneal epithelial proliferation was observed in CD25-deficient mice, a model of Sjögren’s syndrome(Stepp et al., 2018b, p. 25). Thus, several rodent models of DED recapitulate the morphological alterations in corneal innervation observed in patients and offer an opportunity to understand how they occur. By contrast, a rat model of DED induced by chemical denervation of the lacrimal gland in which there is altered tear protein composition but no reduction in basal tear production showed no changes in ICN(Aicher et al., 2015; Hegarty et al., 2018). Table 2 summarizes the numerous animal studies on the subject.

The causes of the morphological changes in ICNs in DED are poorly understood, but they are likely related to the same factors that bring about corneal epitheliopathy because of the intimate contact between corneal nerves and epithelial cells(Stepp et al., 2017). Tear hyperosmolarity, a core pathophysiological mechanism, readily induces ICN degeneration in mice(Guzmán et al., 2020) and rats(Hirata et al., 2015). DED patients have reduced corneal epithelial thickness and cell density throughout the epithelial cell layer, most likely resulting from increased desquamation, inflammatory apoptosis, and impaired regeneration (Simsek et al., 2018). The purely mechanical effect of increased attrition in DED might also be relevant(Stepp et al., 2018a). Therefore, as ICNs are shed along with corneal epithelial cells(Hegarty et al., 2017), the changes in corneal nerve morphology might be caused by the altered corneal epithelial cell physiology in DED. Supporting the existence of this close link between corneal epithelial cells and ICNs, mouse strains with higher corneal nerve density exhibit faster corneal epithelial wound repair(Pham et al., 2019). In addition, ICNs are closely associated with intraepithelial dendritic cells, and the two are functionally interdependent(Gao et al., 2016; Hamrah et al., 2016). Thus, considering the role of dendritic cells as sentinels of the immune response and the numerous changes that DED brings about in these cells(Guzmán et al., 2020; Jamali et al., 2020; Senthil et al., 2021; Yamaguchi et al., 2016; Zheng et al., 2010), it is possible that corneal nerve-dendritic cell crosstalk also contributes to the morphological changes in corneal nerves. More research is warranted on the mechanisms that cause corneal nerve damage in DED.

### 4.2 Changes in corneal nerve physiology

As is the case for morphological abnormalities, there is also ample clinical evidence of somatosensory dysfunction of corneal nerves in DED [see reviews(Guerrero-Moreno et al., 2020)]. Of the two methods available for corneal sensitivity testing in patients, the Cochet-Bonnet aesthesiometer is simpler and more widely used than the Belmonte aesthesiometer (Golebiowski et al., 2011). The Cochet-Bonnet aesthesiometer is based on mechanical stimulation, which only effectively activates the Aδ selective mechano-nociceptors found among the ICBNs. With this method, a reduction in corneal mechanical sensitivity (hypoesthesia) has been reported in DED that correlates with the decreased density of ICBNs observed by in vivo confocal microscopy(Labbé et al., 2012, 2013) and that improves with cyclosporine A (immunomodulatory) treatment(Toker and Asfuroğlu, 2010). By contrast, the non-contact Belmonte aesthesiometer can apply mechanical, chemical, and physical stimuli, potentially differentiating between selective mechano-nociceptors, polymodal nociceptors, and cold thermoreceptors. With this method, some studies found decreased corneal sensitivity to all three modalities in DED that correlated with morphological nerve abnormalities and disease severity(Benítez-Del-Castillo et al., 2007; Bourcier et al., 2005). Contrastingly, others reported increased mechanical and chemical sensitivity (hyperesthesia) that also correlated with corneal staining or other severity indicators(De Paiva and Pflug-felder, 2004; Situ et al., 2008; Spierer et al., 2016; Tuisku et al., 2008). These contradictory findings could stem from differences in the model of the Belmonte aesthesiometer used and the considerable heterogeneity of DED patients. In line with this, another study reported reduced corneal mechanical sensitivity by both contact and non-contact aesthesiometry in aqueous-deficient DED but not in meibomian gland dysfunction DED patients(Rahman et al., 2015). A large, more recent study showed heterogeneity in corneal mechanical sensitivity in DED patients(Galor et al., 2021): 13% of cases had reduced mechanical thresholds (hyperesthesia) that were associated with worse symptoms and 10% of cases had increased mechanical thresholds (hypoesthesia) that were associated with worse corneal epitheliopathy. Also of note is the fact that sustained tear instability in normal subjects affects corneal sensitivity to different modalities: sensitivity decreases with cold stimulation but increases with mechanical and chemical stimuli; this effect lasts for at least 30 min (Awisi-Gyau et al., 2019; Situ et al., 2019). Thus, the rapid adaptive capacity of corneal nerves to changing environmental conditions could also confound measurements.

There is an alternative hypothesis to explain the discrepancy in clinical corneal somatosensory findings in DED. At first, corneal nerve damage in DED might uniformly lead to corneal mechanical hypoesthesia, but in some cases, dysregulated compensatory mechanisms acting both in the corneal nerves and in the central nervous system could lead to peripheral and central sensitization, respectively, with corneal mechanical hyperesthesia thereby ensuing(Crane et al., 2017; Spierer et al., 2016). Interestingly, hypersensitivity of corneal cold thermoreceptors was reported in patients with recent-onset DED but not in those with long-standing disease(Corcoran et al., 2017). Furthermore, corneal sensitivity to capsaicin, a TRPV1 agonist, varied markedly with DED type in another study(Kaido et al., 2020). Consequently, not all sensory modalities are affected equally in DED. In addition, neuropathic pain, i. e. that derived from changes in the somatosensory nervous system, could be considered a maladaptive response to corneal nerve damage(Costigan et al., 2009). A subset of DED patients show no signs of corneal epitheliopathy but report ocular pain, which is regarded as neuropathic (Crane et al., 2017; Kaido et al., 2016). In some of these patients, topical anesthesia (which impairs conductivity of corneal nerves) reduces symptoms, suggesting that peripheral sensitization plays a role in ocular pain. In others, it has no effect, evidencing the contribution of central sensitization to ocular pain, and consistently, these patients have a lower threshold for evoked pain in extraocular locations (forehead and forearm) (Crane et al., 2017).

Animal studies on corneal nerve function in DED also show disparate results (Table 3). Using a modified Cochet-Bonnet esthesiometer on the murine desiccating stress model, Stepp et al. reported a quick drop in corneal mechanical sensitivity that is sustained for at least 10 days (Stepp et al., 2018a), and Simsek et al. reported similar findings with a different mouse strain after 2 and 4 weeks(Simsek et al., 2019). Like-wise, Yamazaki et al. observed reduced mechanical sensitivity in mice after 4 weeks of disease induction by extraorbital lacrimal gland excision (Yamazaki et al., 2017). Chemical denervation of the lacrimal gland in rats only affected tears qualitatively, which led to reduced corneal sensitivity to menthol (TRPM8 agonist) but not to capsaicin(Aicher et al., 2015) and reduced blinking(Hegarty et al., 2018). By contrast, a rapid but reversible increase in corneal mechanical sensitivity was reported in a sleep deprivation-induced model of murine DED(Li et al., 2018). A sustained increase was observed in mice 1–3 weeks after extra- and intraorbital lacrimal gland removal(Fakih et al., 2019). In the latter study, mice also exhibited concomitant signs of spontaneous eye pain and neuroinflammatory changes in the trigeminal ganglion and the brainstem, evidence of central sensitization(Fakih et al., 2019). Similarly, another group observed corneal mechanical hypersensitivity in rats after 1 week of lacrimal gland removal(Meng et al., 2015), and we reported hypersensitivity to hypertonicity in mice exposed to hyperosmotic stress for 5 days(Guzmán et al., 2020). DED also modified the responses to cooling, menthol, noxious heat, and capsaicin of cornea-innervating neurons in rats, with sensitization followed by prolonged desensitization to stimulation reported after 1 and 8 weeks of lacrimal gland removal(Hatta et al., 2019; Kurose and Meng, 2013). Remarkably, an increase in the fraction of trigeminal neurons coexpressing TRPM8 and TRPV1 and a reduction in those expressing only TRPV1 were observed in the same animals and might explain the functional changes(Hatta et al., 2019). In guinea pigs, chronic DED heightened TRPV1-dependent responses in both cold-sensitive and cold-insensitive corneal nerves(Masuoka et al., 2020).

In summary, some animal models show decreased corneal mechanical sensitivity while others exhibit reduced mechanical thresholds (increased sensitivity). Similarly, opposite findings for cold thermoreceptor and polymodal nociceptor-mediated responses have been reported as well. In addition to the inherent complexity and variation of physiological measurements, sex, species, and strain differences in corneal innervation might account for some of the discrepancies in these studies(Pham et al., 2019). Unsurprisingly, this heterogeneous picture of corneal nerve physiology in DED derived from animal studies is consistent with the somewhat contradictory reports on corneal nerve physiology in DED patients summarized above. The lack of a unifying model of corneal nerve dysfunction in DED likely represents the diverse presentations of the disease, with varying degrees of direct neural damage and compensatory responses.

### 4.3 Corneal nerve dysfunction, tear production and blinking

Basal tear production decreases in aqueous-deficient DED, which constitutes a defining clinical finding for diagnosis(Willcox et al., 2017), and as explained above, TRPM8 activation in corneal cold thermoreceptors sustains basal tear production through a neural arc(Parra et al., 2010). Although intrinsic immune-mediated defects in lacrimal gland function are responsible for reduced tear production in some patients, the contribution of corneal nerve dysfunction impairing the afferent arm of the tearing neural arc cannot be ignored(Meng and Kurose, 2013). Supporting this notion, topical menthol combined with heat markedly increased tear volume in aqueous-deficient DED patients in one clinical trial(Arita et al., 2017), although another study did not observe such effect with menthol alone(Kovács et al., 2016b). However, the latter study reported alleviation of basal unpleasant symptoms in DED patients, which was interpreted as further evidence of dysfunctional corneal TRPM8-mediated signaling in this group. Studies in rats and guinea pigs show that DED affects the thermal and menthol-induced responses of corneal cold thermoreceptors(Kovács et al., 2016b, 2016a; Kurose and Meng, 2013), leading to hyperexcitability and enhanced desensitization post-stimulation. Interestingly, the abnormal activity in the guinea pig model is restricted to corneal cold thermoreceptors, with little change observed in polymodal nociceptors and selective mechanoreceptors(Kovács et al., 2016b). In mice, non-DED-related (surgically induced) corneal nerve injury upregulates TRPM8 expression, cold- and menthol sensitivity, and the ongoing firing activity of corneal cold thermoreceptors, leading to increased basal tear production(Piña et al., 2019). Of note, age is a significant risk factor for DED. In one study(Alcalde et al., 2018), aged mice displayed changes in the architecture (branching) and TRPM8 expression levels of ICN and abnormal cold- and menthol-evoked firing responses of corneal cold thermoreceptors, all of which developed along with increased basal tear production and tear osmolarity. In humans, aging brings about a reduction in density(Niederer et al., 2007; Roszkowska et al., 2021) and increased tortuosity(Chirapapaisan et al., 2021) of ICN and decreased corneal mechanical sensitivity(Mirzajan et al., 2015; Roszkowska et al., 2004). Thus, changes in corneal sensory input to the tearing neural arc due to corneal nerve dysfunction possibly contribute to altered tear film homeostasis in DED.

Blinking is yet another homeostatic mechanism of the ocular surface that is affected in DED(Belmonte et al., 2017; Rodriguez et al., 2018). Spontaneous blink rate is increased in patients with aqueous deficient DED but not in those with meibomian gland dysfunction (Rahman et al., 2015). Blink rate also increases in animal models of DED(Kovács et al., 2016b; Meng et al., 2015). DED patients present other abnormalities in their spontaneous blink patterns, such as prolonged eyelid closed time and partial blinks(Su et al., 2018). Blinking changes are considered both a cause and a consequence of DED(Belmonte et al., 2017). For instance, the reduced blink rate of video display terminal users in low-humidity environments favors tear film evaporation(Cardona et al., 2011; Himebaugh et al., 2009). Consistently, rats forced to gaze at a fixed spot for 8 h/day under low humidity conditions display reduced blinking and develop DED(Nakamura, 2018), and exposure to tear hyperosmolarity readily induces ICN degeneration in mice(Guzmán et al., 2020) and rats (Hirata et al., 2015). However, increased blinking is associated with tear instability, as tear break-up and corneal exposure serve as blink reflex triggers(Rodriguez et al., 2018). Increased blinking also correlates with decreased corneal mechanical sensitivity in DED patients(Rahman et al., 2015). The link between increased blinking and corneal nerve somato-sensory dysfunction might stem from the fact that ocular surface wetness is sensed by corneal TRPM8+ cold thermoreceptors(Parra et al., 2010), and accessorily, by hyperosmolarity-activated corneal polymodal receptors(Hirata and Meng, 2010; Hirata and Oshinsky, 2012). In line with this, TRPM8-deficient mice have a reduced spontaneous blink rate (Quallo et al., 2015). DED patients show abnormal responses to menthol that vary with disease duration(Corcoran et al., 2017), and this is reproduced in guinea pigs with DED(Kovács et al., 2016b). Remarkably, aged mice show morphological and functional disturbances in corneal TRPM8+ sensory fibers that relate to changes in basal tearing and tear osmolarity(Alcalde et al., 2018), suggesting that abnormal corneal TRPM8-dependent sensory input contributes to age-related DED.

Overall, the published evidence shows that corneal nerve dysfunction in DED is associated with alterations in basal tearing and blinking, two neural arcs that are fundamental for ocular surface homeostasis. But at the same time, there seems to be no simple cause-effect relationship between the two, highlighting the complexity of DED pathophysiology.

## 5 Corneal nerves, pain, and dry eye

Corneal nerves convey the sensory input underlying pain and other abnormal ocular sensations that contribute significantly to the decreased quality of life of DED patients(Belmonte et al., 2017; Gomes and Santo, 2019). The recently revised consensus definition of pain is “an unpleasant sensory and emotional experience associated with, or resembling that associated with, actual or potential tissue damage(Raja et al., 2020).” Pain (the conscious experience) may be classified as nociceptive, neuropathic, or nociplastic. Nociceptive pain arises from actual or threatened damage to non-neural tissue, is usually transient, and due to the activation of nociceptors(Galor et al., 2018; Raja et al., 2020). By contrast, neuropathic pain is caused by a lesion or disease of the somatosensory nervous system, i.e. corneal nerves in the case of the eye(Galor et al., 2018; Raja et al., 2020). More recently, nociplastic pain has been recognized as “that arising from altered nociception despite no evidence of actual or threatened tissue damage causing the activation of nociceptors or evidence of neural lesion or disease(Kosek et al., 2016; Raja et al., 2020).” Of note, the three types of pain are present in the DED clinical spectrum. In addition, the perception of pain is not static, as sensory neurons exhibit plasticity in the form of sensitization and desensitization (Hucho and Levine, 2007). Here we will only provide introductory concepts on the subject. For in-depth information, we refer the reader to excellent reviews on corneal pain and sensation(Belmonte, 2019; Galor et al., 2018; Goyal and Hamrah, 2016).

Nociceptive pain in DED results from high-intensity stimulation of nociceptors(Belmonte et al., 2017). Corneal cell damage and inflammation release soluble mediators that activate corneal polymodal nociceptors through TRPV1 and TRPA1 channels(M. C. Acosta et al., 2013; González-González et al., 2017; Immke and Gavva, 2006; McMahon and Wood, 2006). Transient tear hyperosmolarity in human volunteers elicits painful sensations comparable to those reported in DED patients(Liu et al., 2009), most likely sensed by TRPV1 activation as shown in rats(Bereiter et al., 2018). Mild mechanical activation of Piezo2+ corneal mechanoreceptors also leads to nociceptive pain (Fernández-Trillo et al., 2020), as most forms of corneal stimulation seem to cause either an unpleasant perception or overt pain depending on the intensity of the stimulus(Acosta et al., 2001; Moulton et al., 2012). As an exception, TRPM8-mediated activation of corneal cold thermoreceptors does not induce nociceptive responses in mice at low concentrations of menthol. However, this stimulus does evoke pain at higher levels probably through a TRPM8-independent mechanism (Robbins et al., 2012). In line with this, corneal TRPM8 activation induced by low-concentration menthol (comparable to that triggered by mild tear osmolarity and/or ocular surface cooling) in healthy human volunteers caused no unpleasantness but did increase basal tearing and blinking rate, as expected(Kovács et al., 2016b).

DED-induced peripheral sensitization of corneal nociceptors, most likely caused by local inflammation, plays a significant role in corneal pain(Spierer et al., 2016). Central sensitization, the main underlying mechanism of nociplastic pain, also seems to contribute to ocular pain in DED patients(Crane et al., 2017, p. 201) and animal models(Fakih et al., 2019) but is beyond the scope of this review(Nijs et al., 2021). In isolated mouse eyes, hyperosmolar conditions immediately increase the firing pattern of corneal cold thermoreceptors but known inflammatory mediators do not modify the response of polymodal nociceptors in the same time frame(Parra et al., 2014). Comparable hyperosmolarity-induced sensitization was observed in rats(Hirata and Rosenblatt, 2014). Guinea pigs with DED show changes in the basal activity of corneal mechanoreceptor and polymodal nociceptors fibers at 1 week of disease induction, providing evidence of peripheral sensitization(Kovács et al., 2016b). In the same model, DED enhanced the excitability and responsiveness of corneal cold thermoreceptors to a greater extent, and the increased spontaneous firing rate of these receptors likely relates to the ocular dryness sensation of DED patients. However, another report using the same guinea pig model only observed sensitization of TRPV1-mediated responses in corneal nerves without changes in the ongoing activity and cooling threshold of cold-sensitive nerves(Masuoka et al., 2020), which could be attributed to methodological differences in measurements. In mice, chronic DED increased corneal nerve responsiveness to cold, heat, mechanical, and chemical stimuli in a TRPV1-dependent manner(Fakih et al., 2021). One mechanism behind this change is DED-induced TRPV1 overexpression in TRPM8+ cold-sensitive corneal fibers, which enhances their response to cold and leads to DED-induced cold allodynia (pain evoked by a normally harmless cold stimulus) (Li et al., 2019). Conversely, TRPM8 blockade reduced inflammatory changes in the cornea and trigeminal ganglion in the same model(Fakih et al., 2020). Of note, increased sensitivity to menthol was also observed in DED patients in one report (Corcoran et al., 2017), while others observed heterogeneity in the subjective perception of the same stimulus(Kaido et al., 2021) or capsaicin-induced pain(Kaido et al., 2020) depending on the presence of corneal epitheliopathy or other clinical findings. Intriguingly, sensitization of corneal mechanoreceptors and polymodal nociceptors but desensitization of cold thermoreceptors was observed in an ocular allergy model of guinea pigs(C. M. Acosta et al., 2013), which contrasts with DED. It is tempting to speculate that DED-associated hyperosmolarity, which is absent in ocular allergy, underlies these differences. In line with this, repeated tear film instability decreased corneal mechanical thresholds while increasing cooling thresholds in healthy human volunteers(Awisi-Gyau et al., 2019), and as previously discussed, brief exposure to tear hyperosmolarity induces detectable changes in ICNs in rats(Hirata et al., 2015) and mice(Guzmán et al., 2020).

Regarding the contribution of neuropathic pain to DED, some of the observed changes in corneal receptor excitability of animal models match those reported in injured sensory neurons(Kovács et al., 2016b), which could thus be regarded as neuropathic sensation. Increased spontaneous firing activity, upregulated TRPM8 expression, and increased basal tearing develop in a murine model of surgically-induced corneal nerve injury(Piña et al., 2019). Likewise, polymodal nociceptor sensitization develops in a similar guinea pig model(Luna et al., 2021), and chronic DED in mice upregulates neuropathic pain-related genes in the trigeminal ganglion (Fakih et al., 2021). In humans, there is considerable overlap between symptom descriptors and anatomical nerve abnormalities among patients with DED and with neuropathic pain elsewhere(Galor et al., 2018). Structural-functional correlations between corneal nerves and symptoms in neuropathic pain patients are much sought after because of their diagnostic potential. However, the issue of whether corneal microneuromas resulting from abnormal nerve regeneration after damage actually do occur in these patients or instead represent a misinterpretation of normal anatomy remains to be settled (Chinnery et al., 2021; Dermer et al., 2021; Moein et al., 2020; Stepp et al., 2020a). Interestingly, the location of action potential initiation in corneal nociceptive fibers and how it shifts towards the stimulus initiation by the effect of pro-inflammatory mediators has been determined in mice(Goldstein et al., 2019). Our increased understanding of how the morphology of nociceptive nerve terminals in the cornea affects signal processing(Barkai et al., 2020) and the ability to image nociceptive channels at work(Aleixandre-Carrera et al., 2021) should lead to a better grasp of neuropathic pain in DED.

In summary, pain and abnormal sensation in DED have both inflammation (i.e. nociceptive) and corneal nerve injury (i.e. neuropathic) as pathophysiological mechanisms(Belmonte, 2019), which contrasts with other ocular surface disorders such as ocular allergy that have an inflammatory basis but no demonstrable corneal nerve damage (Royer et al., 2019).

## 6 Conclusion

**Fig. 3:**
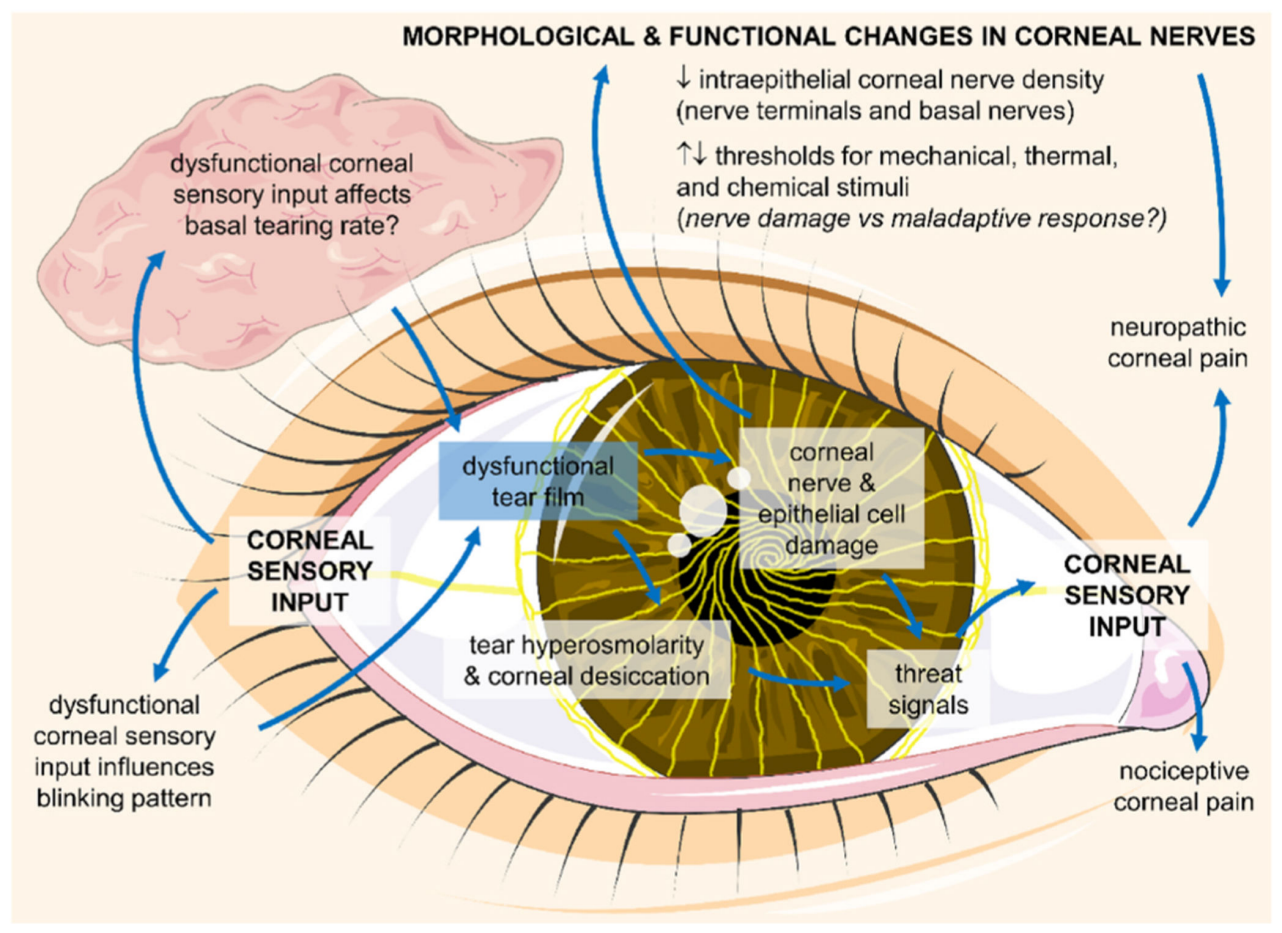
Summary of the roles that corneal nerves play in dry eye pathophysiology. A dysfunctional tear film, the defining feature of the disease, leads to tear hyperosmolarity and corneal desiccation, which are associated with corneal nerve and epithelial cell damage. A wide range of morphological and functional changes in corneal nerves has been described. Corneal nerve damage itself is the source of sensory input linked to neuropathic pain, whereas the threat signals that are released upon ocular surface damage are sensed by corneal nerves and lead to nociceptive pain. In addition, dysfunctional corneal sensory input affects basal tearing rate and blinking patterns, which fuel the vicious cycle of the disease and favor its progression.

Corneal nerves, as integral components of the cornea, are naturally involved in the pathophysiology of DED (Fig. 3). Although this may seem superfluous today, it was not so evident to many researchers some years ago. There is a stark divide in the scientific literature around neuroscientific and non-neuroscientific strategies for the study of this prevalent ocular surface disorder, a tendency that fortunately has started to turn in recent times. A more comprehensive approach to the exploration of DED, one that encompasses both neural and immunological aspects of its pathogenesis, should result in a better understanding of the most impactful symptoms from the patients’ perspective. Conversely, the neuro-inclusive view of DED is not just about pain and unpleasant sensation because corneal nerves constitute pillars of ocular surface homeostasis, as summarized in this review. Therefore, we believe that much is to be gained from bridging both sides in the DED research field.

## Figures and Tables

**Table 1:** Animal studies on functional profiles and ion channel expression of corneal trigeminal neurons and nerve fibers.

Report - First author (year)	Species	Type of study	Findings
Jiao et al. (2021)	Mouse	Corneal nerve fibers	40% TRPV1+
González-González et al. (2017)	Mouse	Corneal nerve fibers	49% cold thermoreceptors, 41% polymodal nociceptors, 10% mechano-nociceptors
Alamri et al. (2015)	Guinea Pig	Corneal trigeminal neurons	45% TRPV1+, 28% Piezo2+, 8% TRPM8+
Bron et al. (2014)	Guinea Pig	Corneal trigeminal neurons	30% Piezo2+, 14% TRPM8+
Ivanusic et al. (2013)	Mouse/Guinea Pig	Corneal trigeminalneurons	18% TRPM8+/12% TRPM8+
Nakamura et al. (2007)	Rat	Corneal trigeminal neurons	23% TRPV1+
Murata and Masuko (2006)	Rat	Corneal trigeminal neurons	37% TRPV1+

**Table 2:** Animal studies on DED-induced morphological changes in corneal nerves.

Report, first author (year)	Species, strain, age, and sex	DED/corneal aggression model	Method	Morphological changes
Simsek et al. (2018)	8-week-old male Balb/c mice	Desiccating environment	In vivo confocal microscopy	↓ ICBN density, no change in tortuosity & reflectivity after 3 days
Simsek et al. (2019)	8-week-old male Balb/c mice	Scopolamine (controlled-release pump)	In vivo confocal microscopy	↓ ICBN density, no change in tortuosity & reflectivity after 4 weeks
Stepp et al. (2018a)	6 to 8-week-old female C57BL/6 mice	Scopolamine + desiccating environment	Confocal microscopy	↓ ICBN & ↓ ICNT density after 3, 5, and 10 days
Stepp et al. (2018b)	4 to 10-week-old mice of both sexes	CD25 null mice (C57BL/6 background), spontaneous Sjogren-syndrome model	Confocal microscopy	↓ ICBN density starting at 4 weeks of age, ↑ ICNT density, ↓ stromal nerve density
Hegarty et al. (2017)	Male Sprague-Dawley rats	Single topical application of menthol, capsaicin, or hypertonic saline	Confocal microscopy	↓ ICN density 2 h after menthol & capsaicin, ↓ ICN density 7 days after capsaicin; ↑ varicosities after capsaicin & hypertonic saline
Guzmán et al. (2020)	6 to 8-week-old Balb/c mice of both sexes	Hypertonic saline instillation 4 times/day for 5 days	Confocal microscopy	↓ ICBN & 1 ICNT density after 5 days
Yamazaki et al. (2017)	9 to 10-week-old C57BL/6 mice	Unilateral extraorbital lacrimal gland excision	Confocal microscopy	↓ ICBN density after 4 weeks
Fakih et al. (2019)	7 to 8-week-old female C57BL/6	Unilateral extraorbital & intraorbital lacrimal gland	Confocal microscopy	↓ ICNT density after 3 weeks
Aicher et al. (2015)	mice 250–450g maleSprague-Dawley rats	excision Chemical denervation of extraorbital lacrimal gland	Confocal microscopy	No change in ICN density after 3–4 weeks
Mecum et al. (2020)	8 to 10-week-old C57BL/6 mice of both sexes	Unilateral extraorbital or extraorbital & intraorbital lacrimal gland excision	Confocal microscopy	No change in ICBN & ICNT density after 2 weeks

**Table 3:** Animal studies on DED-induced changes in corneal nerve function.

Report - First author (year)	Species, strain, age, and sex	DED model	Method	Functional changes
Simsek et al. (2019)	8-week-old male Balb/c mice	Scopolamine (controlled-release pump)	Modified Cochet-Bonnet	↓ mechanical sensitivity after 2 and 4 weeks
Stepp et al. (2018a)	6 to 8-week-old female C57BL/6 mice	Scopolamine + desiccating environment	Modified Cochet-Bonnet	↓ mechanical sensitivity after 3, 5, and 10 days
Stepp et al. (2018b)	4 to 10-week-old mice of both sexes	CD25 null mice (C57BL/6 background), spontaneous Sjogren-syndrome model	Modified Cochet-Bonnet	↓ mechanical sensitivity after 6 weeks of age
Guzmán et al. (2020)	6 to 8-week-old Balb/c mice of both sexes	Hypertonic saline instillation 4 times/day for 5 days	Hypertonic stimulus	↑ blinking response after 5 days
Yamazaki et al. (2017)	9 to 10-week-old C57BL/6 mice	Extraorbital lacrimal gland excision	Modified Cochet-Bonnet	↓ mechanical sensitivity after 4 weeks
Fakih et al. (2019)	7 to 8-week-old female C57BL/6 mice	Unilateral extraorbital & intraorbital lacrimal gland excision	Spontaneous eye closing ratio; von Frey filaments	↓ eye opening after 1, 2, and 3 weeks ↑mechanical sensitivity after 1, 2, and 3 weeks
Aicher et al. (2015)	250–450g male Sprague-Dawley rats	Chemical denervation of extraorbital lacrimal gland	Menthol & capsaicin stimuli	↓ sensitivity to menthol but not to capsaicin after 3–4 weeks
Mecum et al. (2020)	8 to 10-week-old C57BL/6 mice of both sexes	Unilateral extraorbital or extraorbital & intraorbital lacrimal gland excision	Eye closing ratio & eye wipes	↓ eye opening and ↑capsaicin-induced wiping after 2 weeks (↑ change in females)
Masuoka et al. (2020)	4-week-old male Hartley guinea pigs	Bilateral extraorbital lacrimal gland removal	Blink rate	↑ spontaneous & capsaicin-induced blinking from weeks 1–4
Hatta et al. (2019)	300–400g male Sprague-Dawley rat	Unilateral extraorbital or extraorbital & intraorbital lacrimal gland excision	Electrophysiological recordings (trigeminal ganglion neurons)	↑ capsaicin-induced basal activity and ↑ capsaicin-induced suppression of cool-evoked activity of corneal cold-sensitive neurons; ↑ co-expression of TRPV1 & TRPM8 in corneal cold-sensitive neurons

## Data Availability

Data will be made available on request.

## Abbreviations

DED: dry eye disease
ICBN: intraepithelial corneal basal nerve
ICNT: intraepithelial corneal nerve terminal
ICN: intraepithelial corneal nerve
TRPV: transient receptor potential vanilloid
TRPA: transient receptor potential ankyrin
TRPM: transient receptor potential melastatin.
